# Salvage double-guidewire-assisted endoscopic ultrasound-guided rendezvous drainage for disrupted pancreatojejunostomy

**DOI:** 10.1055/a-2955-9759

**Published:** 2026-09-30

**Authors:** Yusuke Yoshikane, Takamitsu Tanaka, Reiko Yamada, Tetsuro Miwata, Kenji Nose, Hayato Nakagawa

**Affiliations:** 1 Department of Gastroenterology and Hepatology 220937 Mie University Graduate School of MedicineTsuMieJapan

For postoperative pancreatic fistula (POPF) after pancreatoduodenectomy (PD), endoscopic retrograde pancreatography (ERP) is the standard therapeutic approach; however, the procedure is often challenging in patients with surgically altered anatomy. Endoscopic ultrasound-guided rendezvous (EUS-RV) has emerged as a salvage option when ERP fails. 1​ Nevertheless, EUS-guided pancreatic duct drainage (EUS-PDD) remains technically demanding. The double-guidewire (DGW) technique has been reported to facilitate EUS-PDD. 2​ ​3​ We report a POPF case successfully treated with DGW-assisted EUS-RV without complications.

A 45-year-old man underwent PD with child’s reconstruction for advanced gastric cancer with pancreatic head invasion. Intraoperatively placed the pancreatic duct stent across the pancreatojejunostomy dislodged, resulting in POPF, initially managed with computed tomography-guided drainage on postoperative day (POD) 4. However, persistent pancreatic fistula due to the disruption of the pancreatojejunostomy was confirmed ( Fig. 1 ). Two attempts at ERP using a double-balloon enteroscope (DBE) for the pancreatic duct stent placement were unsuccessful ( **Fig.** 2 **and** 3 ). Therefore, EUS-guided pancreatic duct drainage was performed ( Video 1 ). The EUS-RV technique, combined with the DBE, was used to ensure the reliable placement of pancreatic duct stents. Additionally, the DGW technique was used to increase the likelihood of successful stent placement.

**Fig. 1:**
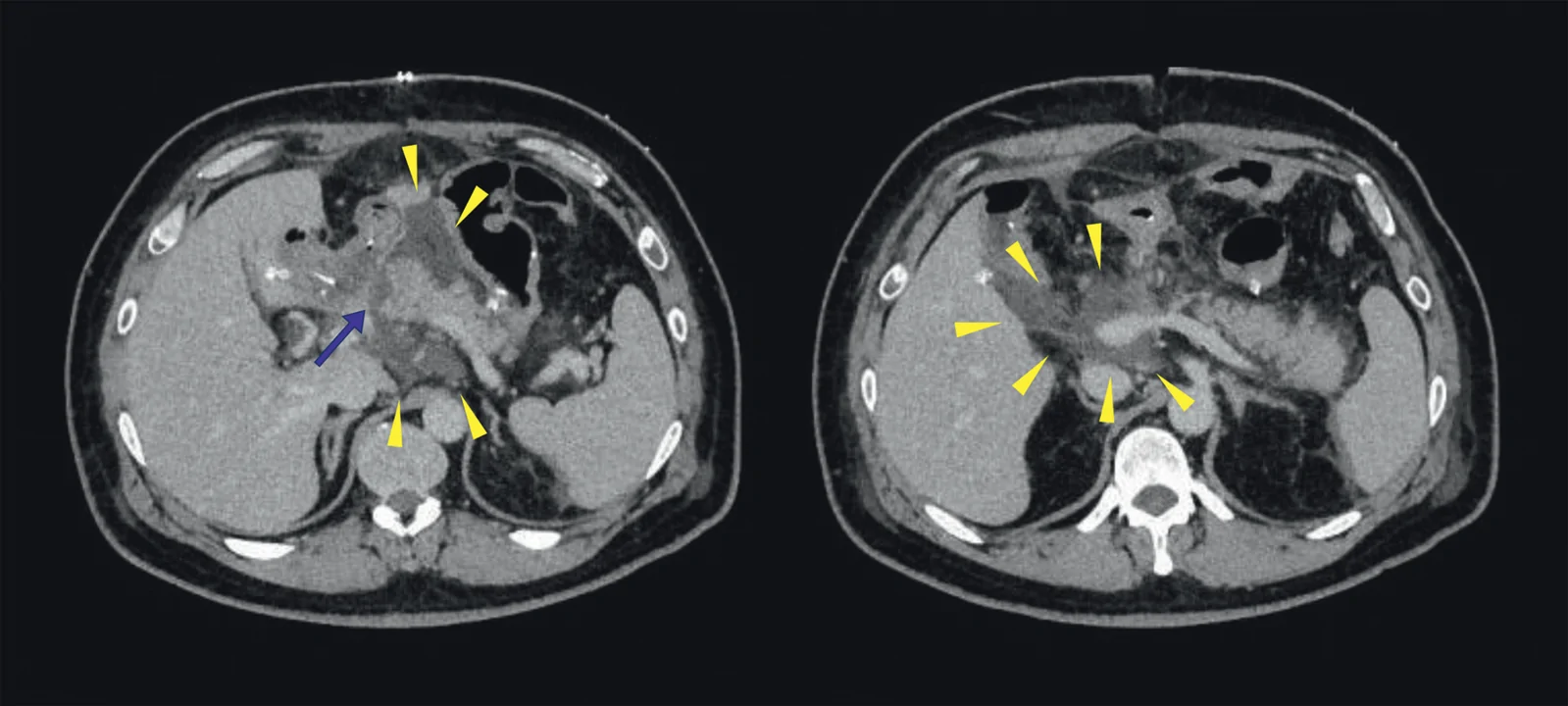
After computed tomography (CT)-guided drainage, a persistent pancreatic fistula (the yellow arrows) due to the disruption of the pancreatojejunostomy (the blue arrows) was confirmed.

**Fig. 2:**
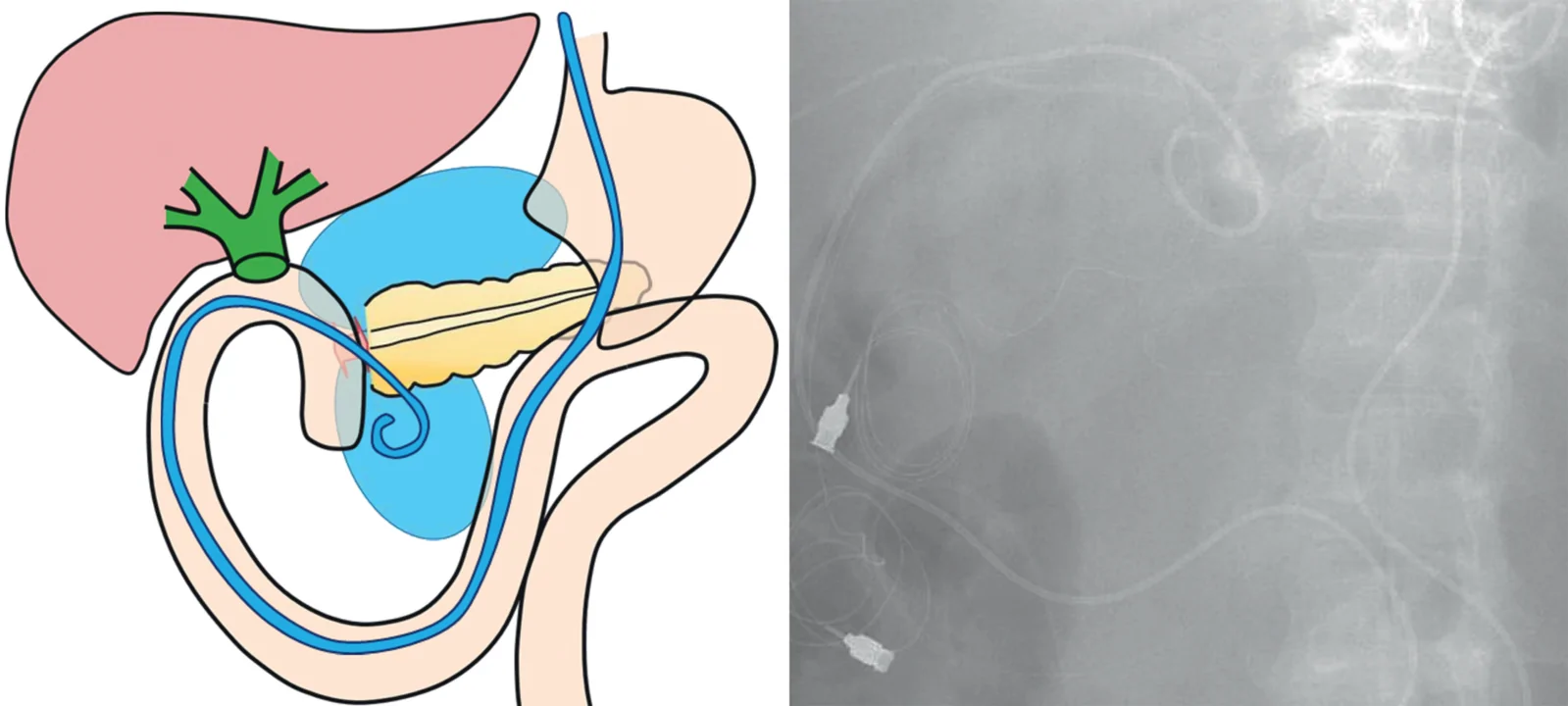
Endoscopic retrograde pancreatography (ERP) using a double-balloon enteroscope (DBE) was attempted but unsuccessful. An endoscopic nasobiliary drainage (ENBD) tube was placed for the fistula drainage.

**Fig. 3:**
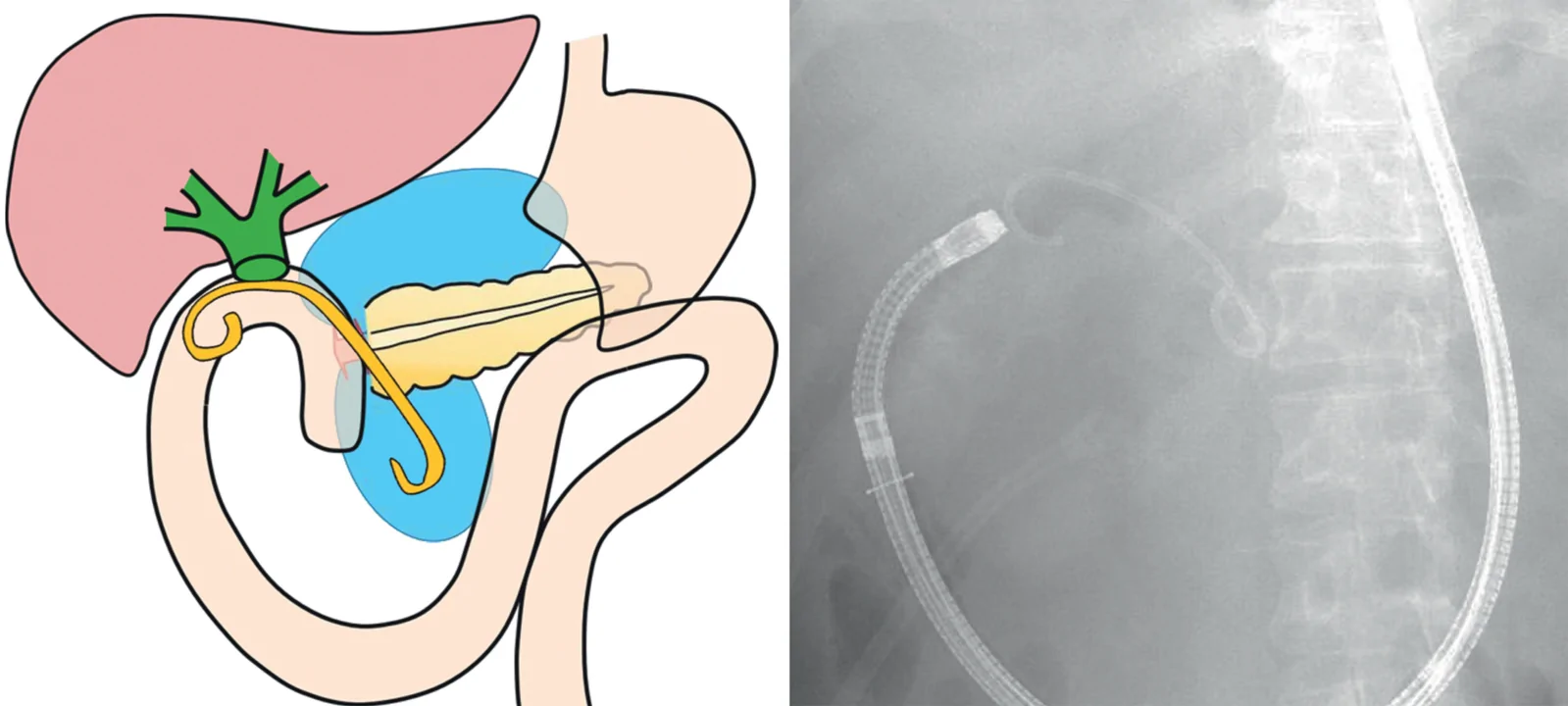
Despite repeated attempts, double-balloon enteroscopy-assisted endoscopic retrograde pancreatography (DBE-ERP) was unsuccessful, and a double-pigtail tube was placed for the fistula drainage.

**Video 1** Double-guidewire–assisted endoscopic ultrasound-guided rendezvous drainage for disrupted pancreatojejunostomy. After transgastric pancreatic duct puncture, two guidewires were advanced using a double-lumen catheter. A pancreatogastrostomy stent was placed, followed by successful transanastomotic pancreatic duct stenting under double-balloon enteroscopy guidance.


EUS-RV was performed on POD 49. The pancreatic duct was punctured transgastrically with a 22-gauge needle, and a 0.018-inch guidewire was advanced. After tract dilation with a drill dilator, a double-lumen catheter was inserted, and a 0.035-inch guidewire was advanced across the pancreatojejunostomy. To prevent pancreatic leakage, a 5-Fr straight stent was placed at the pancreatogastrostomy. Transanastomotic stenting was then performed under DBE guidance, enabling the direct visualization of the disrupted anastomosis and secure access for reintervention ( Fig. 4 ).

**Fig. 4:**
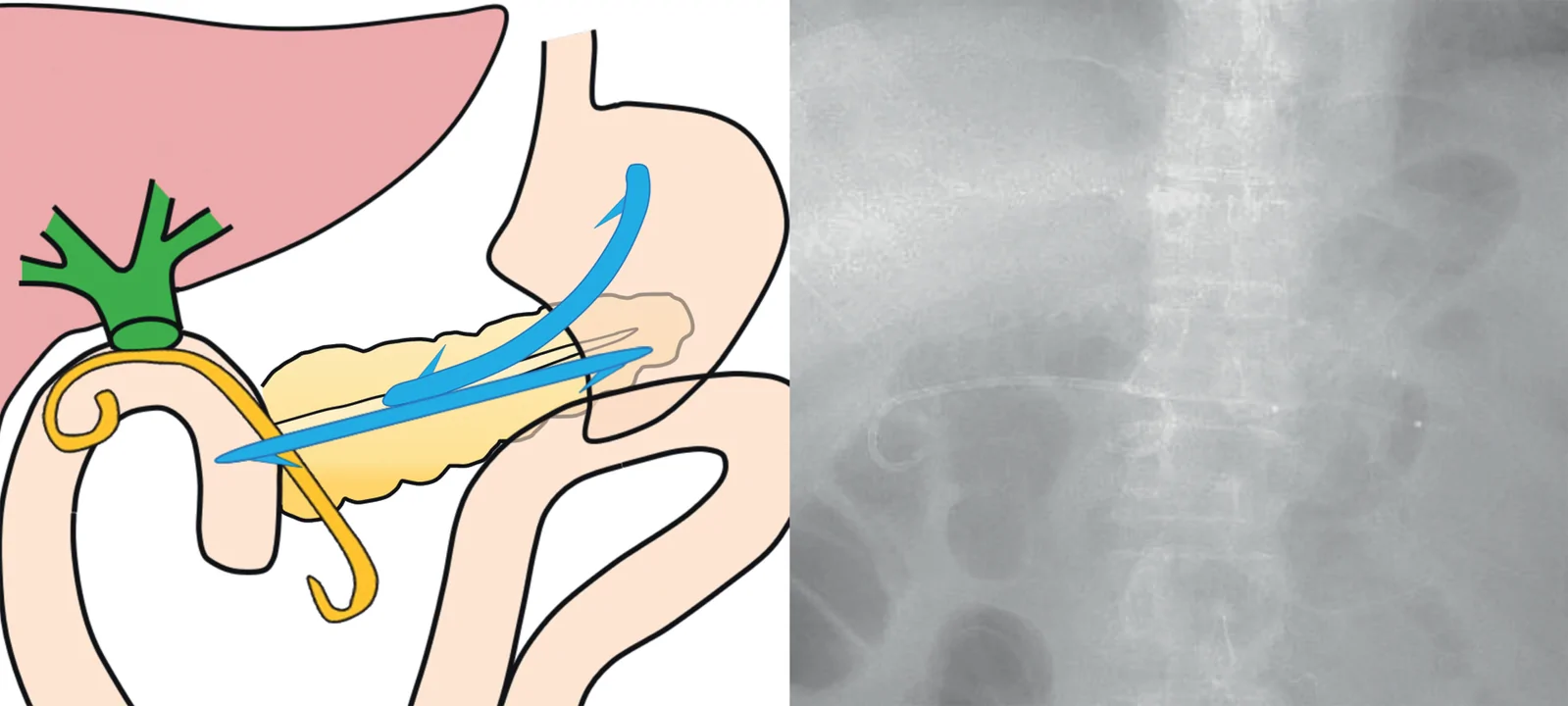
Finally, two pancreatic duct stents were placed after the double guidewire (DGW)-assisted endoscopic ultrasound-guided rendezvous (EUS-RV) drainage.

At 6-month follow-up, the transanastomotic stent was upsized from 5-Fr to 7-Fr, and the pancreatogastrostomy stent was removed without adverse events.

The DGW-assisted approach may facilitate precise, safe anastomotic access and effective long-term drainage.

Endoscopy_UCTN_Code_TTT_1AS_2AK

## Declaration of GenAI Use

The authors confirm that no generative artificial intelligence was used in the preparation of this manuscript.
